# Measuring optic nerve sheath diameter using ultrasonography for the detection of non invasive intracranial pressure: what it is and what it is not

**DOI:** 10.1590/0004-282X-ANP-2022-E006

**Published:** 2022-08-08

**Authors:** Chiara Robba

**Affiliations:** 1 San Martino Policlinico Hospital, Anesthesia and Critical Care, IRCCS for Oncology and Neuroscience, Genoa, Italy.; 2 University of Genoa, Department of Surgical Sciences and Integrated Diagnostics, Genoa, Italy.

Over the last decade, the study of optic nerve sheath diameter (ONSD) using ultrasonography has gained particular interest for the assessment of non-invasive intracranial pressure (nICP)^1^. 

ONSD ultrasonography is an elegant technique, which has the advantage of being easily available, repeatable at bedside, low cost and cheap, avoiding the risks of infection or hemorrhage related to intracranial catheters. The pathophysiology behind this technique is simple: the optic nerve is surrounded by the meninges and the subarachnoid space, and has elastic properties; therefore, when intracranial pressure (ICP) rises, the optic nerve sheath is distended and the ONSD increases.

ONSD has been evaluated on Magnetic Resonance imaging and Computed Tomography studies^2^ ^,^ ^3^, demonstrating a good accuracy for the real time estimation of ICP. The possibility to evaluate ONSD as a surrogate of ICP using a safe method, which does not require the transfer of patients to the radiological suite and does not expose them to ionizing radiation has led to a high number of studies and publications on the Ultrasound based estimation of this tool. 

In general, observational studies and meta-analysis suggest promising results regarding the correlation between ONSD and nICP, showing moderate to high sensitivity for the detection of elevated ICP (86% to 97%)^4^ ^-^ ^6^.

However, some striking points and issues have been described for this tool, especially regarding the lack of a universal methodology used in different studies. A vast heterogeneity is present in the literature regarding the transducers and frequencies adopted^4^ ^,^ ^5^ by researchers, the measurements planes^4^ ^,^ ^5^, the number of measures from one or two ONSDs, the patient´s positioning^4^ ^,^ ^5^; in addition, a non-universal definition of the quality of the images obtained and of the US visualization of ONSD exists, thus resulting in the measure in some cases of the ONS instead of the entire ONSD^4^ ^,^ ^5^.

Open questions also remain, such as the ability of ONSD to return to its initial size after treatment or resolution of increased ICP, or in case of intracranial hypotension. 

In patients after subarachnoid hemorrhage (SAH), for instance, dramatic increases of ICP consequent to aneurysm rupture can lead to a disruption of the elastic properties of the ONS membrane, thus resulting in enlarged ONSD even without the presence of intracranial hypertension^7^. 

In addition, a major issue remains the large variations in ONSD cutoffs evaluated to estimate the critical threshold of ICP> 20 mmHg, ranging from 4.2 to 6.5 mm with wide confidence intervals according to literature^8^ ^,^ ^9^. 

Systematic reviews and meta-analysis^5^ ^,^ ^9^, suggest the best cut-off value of ONSD of 5.1 and 5.8 mm, with the area under the hierarchical summary receiver-operating characteristic curve of ONSD for predicting increased ICP of 0.938^9^. 

Studies on healthy volunteers show also heterogeneity on the normal values of ONSD according to ex, age and race^10^. 

 In a study conducted in China, the mean ONSD value was 4.33 ± 0.38 mm in normal individuals and 6.61 ± 0.39 mm in patients with increased ICP^10^. 

In healthy volunteers in Europe^11^, ONSD was significantly different between males and females [4.2 (3.9-4.6) mm vs. 4.1 (3.6-4.2) mm, *p*= 0.01)] and it was correlated with age, with increasing values in the elderly population (R = 0.50,*p*< 0.0001). However, in traumatic brain injured patients, no differences in ONSD were found according to sex and age, thus suggesting that different ONSD cut-off values do not need to be age- or sex-adjusted in brain injured patients patients.

Finally, a possible inter-intraobserver variability has been reported, which is an intrinsic limitation of US technique^12^. 

Potentially, well-defined criteria for training and the definition of educational projects aimed to standardize the methodology of ONSD measurement can importantly minimize these limitations; however, at present, the use of ONSD is limited to the settings of specialized Neurocritical Care Units.

 In a recent consensus of experts of the European Society of Intensive Care, ONSD was not considered as a basic skill for general intensivists^13^; this was related to the idea of nICP estimation as an advanced skill with no consideration of this tool in the general intensive care unit training and certifications programs. 

However, a consensus of experts considering only the neurocritical care settings defined ONSD as a “basic-plus skill”, which requires training and an appropriate learning curve, but which should be considered fundamental for the management of these patients^14^. This suggests the need of implementing brain ultrasonography in a process of formal certification processes, consensus statements, and documents also outside the neurocritical care settings to ensure the widespread use of this technique. 

All these limitations have led to the concept that ONSD cannot provide a value of ICP “as a number”, thus making it unfeasible and not accurate enough to be used to substitute invasive ICP measurement, especially in brain injured patients. 

However, ONSD has still to be considered a valuable technique in other clinical contexts. 

In a recent study, ONSD was measured in patients with Idiopathic intracranial hypertension (IIH) and compared to normal healthy individuals^15^.

Ninety-seven participants aged 18-80 years were divided into two groups as patients with IIH (n=47) and the control group (n=50). The mean ONSD was statistically significantly larger in the IIH group compared to the control group (6.4 vs 4.90 mm). The cut-off value of ONSD in patients with IIH was measured as 5.70 mm. Also, a positive correlation between body mass index and ONSD (r= 0.437, p<0.001) was found.

This study clearly represents one of the most useful applications of ONSD. Although the cut-off is not completely determined in literature, the difference of median ONSD found between the two groups suggests that these patients, taking in consideration age, sex, and BMI, ONSD can reliably help in the qualitative discrimination of high vs low ICP values, and posing the question in borderline situations of the need for additional evaluations, minimizing the risk related to invasive measurements. 

In conclusion, a number of limitations have been demonstrated for the evaluation of ONSD as a surrogate of ICP; despite these limitations have to be taken in consideration, ONSD can be a useful qualitative method to assess the risk of increased ICP, and in particular the changes of ICP within time. This can pave the way on the utilization of this tool in all these situations when invasive tools are not available or contraindicated, but where ICP estimation would be helpful for patient management. This includes a number of neurological conditions (such as meningitis, IIH), or in the general ICU population with no primarily brain injury but with high risk for increased ICP (cardiac arrest, sepsis, pregnancy-related complications etc.). 

In the next years educational projects and research should focus in the standardization of ONSD measurement and training and in the implementation of this method at bedside in the context of a “head to toes” Ultrasound evaluation of patients. 
